# Task-aligned outcome learning in psychiatry: reducing endpoint dilution

**DOI:** 10.3389/fpsyt.2026.1832978

**Published:** 2026-05-11

**Authors:** Eric V. Strobl

**Affiliations:** 1 Department of Biomedical Informatics, University of Pittsburgh, Pittsburgh, PA, United States; 2 Department of Psychiatry, University of Pittsburgh, Pittsburgh, PA, United States

**Keywords:** clinical trials, machine learning, outcome learning, prediction, psychiatry, symptom heterogeneity

## Abstract

Psychiatric research relies on well-defined outcomes for standardization, comparability, and replication, yet investigators often fix broad endpoints before knowing which symptom domains carry task-relevant signal. Even when psychometrically sound and clinically useful, composite measures can dilute predictive information and attenuate treatment effects when predictability or responsiveness concentrates in only a subset of symptoms—thus making studies appear negative despite meaningful change. This Perspective proposes a task-aligned, two-stage machine-learning framework for learning the appropriate outcome. In the first stage, constrained discovery derives a clinically interpretable outcome from a prespecified item pool. In the second, confirmatory evaluation tests the prespecified hypothesis either on a fixed learned outcome, when the aim is to assess a previously derived endpoint in a closely matched study, or on a relearned outcome generated by the same prespecified procedure, when the aim is to test whether that procedure can recover a task-aligned endpoint across different studies. The framework complements psychometrics and open-science practices, shifting focus from broad unsupervised composites to empirically supported targets, with safeguards to keep results interpretable and rigorous.

## 1 Introduction

Psychiatric trials and prediction studies often fall short despite better measurement, larger datasets, and more sophisticated models. Investigators often blame placebo response (1), clinical heterogeneity (2), low power (3), or weak interventions (4). These factors matter, but they can obscure a simpler source of signal loss: researchers often commit to broad endpoints before they know which symptom domains are most predictable or most likely to change with treatment. Composite totals and diagnostic labels can be clinically useful and psychometrically defensible, yet they often combine symptom dimensions that differ in predictability, treatment responsiveness, and mechanistic relevance (5–7).

Consider for example a bupropion trial that prespecifies the Quick Inventory of Depressive Symptomatology–Self Report (QIDS-SR) total score as its primary endpoint, even though the scale includes opposite poles of core symptoms (appetite/weight gain vs loss; hypersomnia vs insomnia) (8). If bupropion improves hypersomnia and increased appetite but worsens insomnia or appetite loss, the total score can mask offsetting symptom shifts and become less sensitive to bupropion’s therapeutic profile (9). This is not an argument to ignore adverse effects; rather, it emphasizes that a single total score can mix therapeutic and adverse shifts into one number and thereby obscure both benefit and tolerability. More generally, broad totals can average targeted improvement with unchanged or counter-directional symptoms, diluting treatment-relevant signal before analysis begins—a problem that extends beyond depression to psychiatric research more broadly.

This Perspective examines the consequences of broad scores across study types. Psychiatry often adopts the same default outcomes for prediction studies, causal analyses, and clinical trials (10, 11). Standardization aids comparability, but poorly aligned endpoints can mask real effects and make “placebo responsiveness” a default explanation for null results (12). I therefore propose a task-aligned machine learning framework, *outcome learning*, to address endpoint dilution. In a constrained discovery stage, investigators learn clinically interpretable outcomes from a prespecified item pool. In confirmatory work, investigators fix whichever feature of the design their hypothesis is meant to test. When the hypothesis concerns a particular previously derived composite, confirmation involves testing that same score in new data. When the hypothesis instead concerns the reproducibility of the learning framework rather than of any single learned endpoint, investigators should hold the learning procedure and its constraints fixed and rederive the outcome within the confirmatory study. This Perspective thus extends rather than challenges existing work on outcome specification (13), outcome reporting bias (14, 15), and estimands (16, 17), all of which underscore the importance of defining confirmatory targets prospectively, reporting them transparently, and minimizing selective outcome reporting.

## 2 Limitations of fixed outcome definitions in psychiatric research

Psychiatry relies on fixed outcomes for good reasons. Standardized scales and diagnostic definitions support communication, interpretation, replication, and meta-analysis (11, 18). The problem is not standardization itself, but what is standardized and when. Psychiatry often fixes both the instrument *and* its scoring rule, usually as a single total score or diagnostic label. Keeping the instrument fixed is often essential, and the instrument may contain clear signals of change. The risk arises when investigators fix a broad scoring rule too early. If symptom domains differ in predictability, treatment responsiveness, or confounding, the total score can average away the very signal the instrument captures. Prediction models may then appear weak even when they predict specific symptom domains well (5), and trials may miss meaningful benefits when an intervention changes only a subset of symptoms that the instrument still measures (6).

The problem worsens when investigators carry the same broad endpoint across tasks with different inferential goals. Prediction studies need stable, learnable targets; mechanistic studies need targets that track the hypothesized pathway; and trials need endpoints that sensitively capture treatment-specific change. A single omnibus outcome rarely serves all three aims well. When researchers nevertheless treat it as a universal target, they can lose power, understate clinically meaningful effects, and draw overly pessimistic conclusions.

These limitations do not imply that fixed outcomes are always misguided, nor do they justify endpoint shopping. The central design choice is *timing*. A more productive workflow keeps the instrument fixed but uses prespecified, constrained discovery to learn a task-relevant, clinically interpretable scoring rule. Confirmatory evaluation should then follow the prespecified hypothesis: if the claim concerns a *particular learned endpoint*, investigators assess that fixed composite directly in new data; if the claim instead concerns the *prespecified procedure* for identifying a task-aligned endpoint, they keep the learning procedure and its constraints fixed and rederive the composite in a new study.

## 3 Prevailing methodological responses and their limitations

Psychiatry has developed several sophisticated responses to the limitations of fixed endpoints, but many target constructs, measurement, models, or sampling rather than the endpoint itself, even when endpoint dilution is the main source of signal loss.

One response focuses on construct. Researchers revise diagnostic categories (19), redefine symptom boundaries (20), and develop alternative nosologic frameworks such as RDoC (21, 22). Related efforts improve measurement through new scales (23), refined item sets (8), repeated assessments (24), multiple informants (25), and psychometric methods such as calibration and harmonization (26). These advances can sharpen constructs and improve reliability, but they often define outcomes without explicit reference to the analytic task. Indeed, psychometric work shows that symptom items and subscales can still differ markedly in the information they provide across severity continua (27, 28), and commonly used assessments continue to aggregate heterogeneous symptom content, allowing summary scores to obscure clinically meaningful variation that may matter differently across tasks (29–31). Yet once such a score has been refined and well validated, investigators often adopt it as a default across prediction, causal, and treatment studies because its psychometric credibility and extensive prior use make it difficult to justify departing from, even when it is poorly matched to the signal a given task is intended to detect.

A second response increases predictor or model complexity while keeping the outcome fixed. Studies add more features, deploy more complex machine learning models, or seek much larger datasets (32). These strategies can help, but they also reduce interpretability (33), raise deployment burden (34), and often weaken transportability across hospitals or health systems (35, 36). When the main limitation lies in the outcome definition, greater model complexity may yield only modest gains.

A third response reduces heterogeneity by restricting the sample. Investigators may recruit narrowly defined subgroups or putative subtypes to create a more homogeneous cohort or enrich for treatment response (37–39). In psychiatry, where randomized trials already raise concerns about external validity and representativeness (40, 41), further restriction can worsen generalizability. In some cases, the need to restrict or cluster patients arises partly because the outcome itself is too broad to capture the relevant signal in a heterogeneous population.

Taken together, these strategies improve constructs, measurements, predictors, or samples while leaving the target outcome largely unchanged. They therefore offer only partial gains when the core problem is mismatch between outcome and task.

## 4 Outcome learning as a solution

Outcome learning takes a different approach by treating psychiatric symptom heterogeneity as a design opportunity. Broad symptom inventories provide a structured item pool of *clinically meaningful variables*, defined here as measured symptoms or functioning domains that correspond to clinically salient aspects of psychopathology, including clinician-recognizable symptoms, subjective distress, and functional impairment. This flexibility matters because investigators often cannot know in advance which symptom domain will be most predictable from baseline data or most responsive to treatment. Prior knowledge may support a broad hypothesis, but it rarely identifies the optimal endpoint with confidence. An intervention such as oxytocin, for example, may not improve social functioning broadly but may affect a narrower domain such as social-emotional reciprocity (42, 43). If a trial prespecifies only a broad endpoint, it may miss a meaningful effect.

Outcome learning addresses this uncertainty by allowing researchers to learn outcomes under prespecified rules and then interpret them clinically after estimation. In the discovery stage, the method does not search freely for favorable results. Instead, it uses a constrained procedure to select, weight, or transform outcome components from a predefined instrument or item pool in a way that matches the task. Constraints such as sparsity, non-negativity, or minimum content coverage can preserve *clinical interpretability*, whereby investigators can inspect and report which items or domains contribute to the learned outcome and in what direction.

Different tasks may justify different outcomes. Prediction studies may benefit from outcomes that emphasize symptom domains that baseline variables can predict reliably. Treatment studies may need outcomes that capture the symptom changes most relevant to the intervention mechanism. Causal analyses may require outcome construction that improves robustness to confounding. In each case, the goal is a clinically interpretable outcome aligned with the scientific objective.

These task-aligned outcomes need not be limited to predefined subscores or single items. In some settings, the most informative target may be an estimated latent symptom dimension derived from a particular combination of items (44). This is especially important in psychiatry, where many phenomena vary along continuous dimensions rather than forming clean subtypes (21, 45). A factor-mixture perspective may better capture this structure by allowing patients to share common latent dimensions while differing in how strongly those dimensions are expressed (46). Outcome learning can build on this view by constructing clinically interpretable outcomes that capture the latent dimensions most relevant to a given task.

Outcome learning can also reduce pressure to escalate predictor-model complexity. When researchers define a clearer target, simpler models may recover meaningful effects and yield more interpretable and generalizable findings without the need to subtype patients (5). Several methodological families already support this approach, including methods that optimize predictability from baseline covariates (5), supervised low-dimensional representations that sharpen treatment separation (9, 47), and composite outcomes for causal identification (6).

## 5 Methodological and inferential considerations

Learning an endpoint rather than fixing a single total score in advance naturally raises questions about validity. Learned outcomes may seem harder to interpret, harder to compare across studies, or more vulnerable to false positives. These concerns are real, but they do not preclude outcome learning. Instead, they require explicit safeguards for outcome construction, as summarized in **Table 1**.

**Table 1:** Common concerns about outcome learning and corresponding safeguards.

Concern	Safeguard
Endpoint shopping	Preregister item pool, constraints, and learning rule; separate discovery from confirmation.
Confirmatory studies require fixed scores	Prespecify the relevant hypothesis; then fix the score or the learning procedure accordingly.
Outcome shifts with predictors/treatments	Treat context-specific learned outcomes as distinct endpoints.
Poor interpretability	Use constrained, inspectable composites (e.g., sparse, nonnegative, or rule-based); report chosen sets, weights, or transforms.
Invalid inference (false positives; overfitting)	Valid inference: permutation/bootstrapping with full refitting; prediction assessed on a held-out test set.
Subjective discounting of selected symptom content	Interpret outcomes at the item level as measured; if content priorities are desired, encode them prospectively via the item pool and constraints.
Not connected to patient-centered relevance	Encode patient-valued domains prospectively through the item pool, minimum content-coverage constraints, or decision-theoretic weights and penalties.
Endpoint proliferation/multiplicity	Learn a prespecified, small set of composite outcomes and apply multiplicity control across them.
Hard to compare across studies or synthesize cumulative evidence	Use a common measurement space and prespecified learning framework across studies; synthesize task-relevant effect estimates with uncertainty; report learned and conventional outcomes.

Outcome learning can preserve rigor and interpretability when treated as a prespecified, constrained component of the analytic pipeline and evaluated with appropriate inference and validation procedures.

Task-aligned outcome learning is not a license for *post hoc* endpoint shopping. It is a prespecified, constrained procedure for defining the study target. Outcome learning is compatible with preregistration, although preregistration is often interpreted as requiring a single fixed endpoint score (13, 18, 48). In discovery-oriented work, preregistration can instead specify the instrument item pool, the clinical constraints on outcome construction, and the algorithm used to derive the outcome for a given task. Confirmatory work can then evaluate replication either by fixing the discovery-derived outcome or by fixing the learning procedure itself. In that sense, outcome learning is not an exception to existing guidance on prespecification, outcome reporting, or estimands, but a proposal for how those principles can be applied when the endpoint is learned from a prespecified item space rather than fixed in advance as a single score (16, 49).

A key nuance is that confirmatory work does not always require investigators to transport a single fixed scoring rule across studies. As in any confirmatory setting, investigators should first prespecify the hypothesis they wish to test and then select the statistical procedure that addresses that hypothesis. In outcome learning, fixing the discovery-derived score is most appropriate when the hypothesis concerns a particular learned composite outcome. For example, in a randomized trial, investigators may ask whether a treatment differs from its comparator on a specific learned endpoint identified previously in exploratory work. This strategy is compelling when the confirmatory study closely matches the exploratory study in comparator, population, setting, and measurement process.

By contrast, relearning the outcome is more appropriate when the hypothesis concerns the prespecified procedure for identifying a task-aligned composite from a common item pool, rather than whether one score should be applied unchanged across contexts. This approach is often preferable when studies differ materially in the comparator set, prediction target, population, or other aspects of context. For example, in prediction research, investigators may hypothesize that a prespecified outcome-learning procedure will yield a clinically meaningful endpoint for risk stratification across studies, without requiring the confirmatory study to reuse the exact composite derived in exploratory work. Likewise, in intervention research, investigators may hypothesize that the same prespecified procedure will recover a clinically meaningful pattern of symptom change across studies, even if the specific learned composite differs between exploratory and confirmatory settings. In such cases, the investigator can prespecify the outcome-learning procedure and its constraints, then apply that procedure within each study to derive a context-appropriate endpoint. What remains fixed prospectively is the rule for constructing the endpoint, not a single invariant scoring function. Replication is then assessed against the relevant hypothesis: that the same prespecified procedure, applied to the same item pool, yields a task-relevant composite outcome across studies, even if its specific features differ by context.

Context dependence of the learned outcome is in fact expected rather than problematic. Learned outcomes may change when the predictor set, treatment, population, or study environment changes: changing predictors changes what can be predicted, changing treatments changes which symptom domains are plausibly affected, and changing populations or study environments can alter which symptom patterns are most informative or most responsive. The safeguard is to make this context dependence explicit—define the task, study population, and study environment; prespecify the candidate item pool and constraints; and treat context-specific learned outcomes as aligned, interpretable endpoints rather than as one universal score. When feasible, reporting both learned and conventional outcomes can further support transparency and comparability.

A separate issue is inference within any given study once the outcome is learned. Outcome learning does not require a rigid divide between exploratory and confirmatory research. When the outcome-learning procedure is prespecified, and when inference properly accounts for the learning step, investigators can make statistically valid within-study claims even if the endpoint itself is learned from the data. However, many familiar tests assume a fixed, prespecified outcome; using them unchanged after outcome learning can yield misleading *p*-values. In randomized studies, valid inference can be obtained with permutation procedures that rerun the *entire* outcome-learning pipeline under each permuted treatment assignment (9, 50). Confidence intervals can be obtained by bootstrap procedures that likewise rerun the full pipeline within each resample. In prediction settings, credible evaluation requires genuine out-of-sample assessment: both the learned outcome and the predictor model should be evaluated on an independent test set (5). Outcome construction should therefore be treated as part of statistical inference, not as preprocessing that can be ignored.

Interpretation also requires care. Investigators may be tempted to discount heavily weighted items by arguing, after the fact, that they reflect side effects rather than therapeutic change or that the selected content is not clinically relevant. Outcome learning should not support such moves. Clinical relevance is specified *a priori* through the choice of instrument and item pool. The instrument defines the measurement space, and the learning procedure reweights observed item responses to optimize a prespecified task under explicit constraints. When particular items receive substantial weight, the appropriate interpretation is literal and task-specific: variation in those measured responses materially contributes to prediction or to differentiating trajectories between study conditions.

This prospective specification should also extend to patient-centered relevance. Statistical optimization alone does not guarantee that a learned composite reflects the symptom domains patients regard as most important. In decision-theoretic terms (51), the learned outcome should not be viewed as the unconstrained maximizer of a purely statistical criterion, but as the solution to a prespecified objective defined over a clinically meaningful item space. Patient priorities can enter that objective prospectively through the choice of item pool, through constraints that require representation of domains such as distress, functioning, or adverse effects, and through weights or penalties that encode the relative importance of different forms of symptom change. Under this view, outcome learning does not infer what should matter from the data alone; rather, it estimates the task-aligned outcome within a value structure specified in advance.

One alternative to outcome learning is to analyze symptom domains or individual items directly rather than learn a composite outcome. Item-level analyses can inform mechanism and safety, but they replace one endpoint with many, creating a substantial multiplicity burden. Even with false discovery rate control, power can fall sharply when effects are modest and dispersed across correlated items, and family-wise error control can become prohibitively conservative (52). Item-level inference also treats questionnaire items as the scientific targets, even though the clinically meaningful quantity is often better viewed as a *latent* outcome that no single item measures perfectly and that may not be captured identically across instruments. In that setting, learning a small number of constrained, clinically interpretable composites offers a pragmatic middle ground: it reduces multiplicity, improves signal-to-noise, and can make clinically meaningful effects more likely to be detected again in later studies than a long list of item-specific effects.

Beyond the interpretation of any single study, context-specific endpoints also raise a broader question about cumulative evidence. Recall that when the hypothesis concerns a prespecified procedure for identifying a task-aligned endpoint, studies need not share the same realized composite to contribute to cumulative evidence. Each study can apply the same outcome-learning procedure within a common measurement space and yield a study-level estimate relevant to the analytic task, together with its uncertainty. Evidence synthesis then combines those estimates rather than the learned item weights or one transported score. The key question is therefore not whether the realized composites are identical, but whether the studies are sufficiently comparable for those estimates to be synthesized meaningfully. Consistent with existing guidance on evidence synthesis (53, 54), synthesis is appropriate only when studies address a sufficiently similar prespecified question and are adequately aligned in population and setting, intervention or predictor information, comparator or intended use, the measurement space and measurement process used to derive the endpoint (including time horizon when relevant), study design, and the effect or performance metric.

## 6 A concrete example with antidepressants

To illustrate outcome learning, we revisit the bupropion example. The question is whether bupropion produces a symptom-change profile that differs from other antidepressants, given its unique activating properties as a norepinephrine–dopamine reuptake inhibitor (55). This possibility is consistent with independent work suggesting that antidepressant effects may vary across symptoms rather than appearing uniformly across total severity scores, even if the literature remains mixed on whether symptom profiles reliably identify the best treatment (31, 56). The results summarized here were previously reported in (9), and we follow the safeguards in **Table 1**. The purpose of this example is not to introduce a new empirical finding, but to illustrate in one concrete setting how the proposed framework links constrained discovery, confirmatory strategy, interpretable endpoint definition, and valid inference.

**Figure 1:**
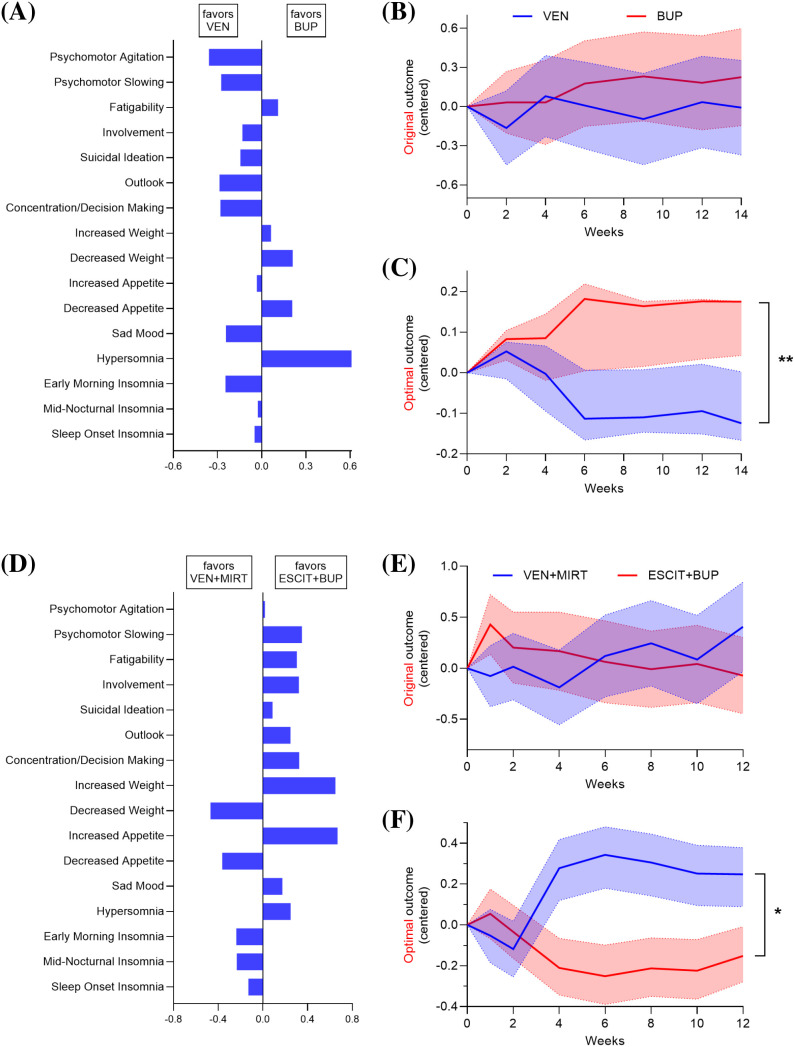
Outcome learning yields interpretable symptom composites with stronger treatment separation in STAR*D and CO-MED. Adapted from (9). **(A)** Symptom weights defining the learned composite in STAR*D Levels 2/2A, shown separately for symptoms favoring bupropion-SR (right) versus venlafaxine-XR (left). **(B)** Longitudinal trajectories in STAR*D using the conventional QIDS-SR total score show substantial overlap; the outcome is mean-centered to highlight between-treatment differences over time. **(C)** Longitudinal trajectories in STAR*D using the learned composite show clear separation, with uncertainty shown via bootstrap confidence intervals; overall separation is assessed using a permutation-based test with family-wise error control. **(D)** Symptom weights defining the learned composite in CO-MED, contrasting escitalopram plus bupropion-SR (right) with venlafaxine-XR plus mirtazapine (left). **(E)** Overlapping longitudinal trajectories in CO-MED using the conventional QIDS-SR total score. **(F)** Clearly separated longitudinal trajectories in CO-MED using the learned composite, with uncertainty from bootstrap resampling; significance is evaluated by permutation testing applied to the full refitted pipeline. *p_FWER_ *<* 0.05, **p_FWER_ *<* 0.01.

We first analyzed Levels 2 and 2A of STAR*D (57) as the constrained *discovery* stage. The item pool was fixed to QIDS-SR items, and the outcome-learning algorithm was prespecified. Patients received bupropion-SR or venlafaxine-XR; sertraline was also included in Level 2 but is omitted here for clarity and was analyzed in (9). STAR*D commonly summarized treatment response with the total score of the 16-item QIDS-SR (58). However, the QIDS-SR total score showed substantial overlap between bupropion-SR and venlafaxine-XR (**Figure 1B**). Historically, clearer evidence for differential effects emerged only through large-scale meta-analysis (59). Our goal, by contrast, is to detect clinically meaningful differential effects earlier, without waiting for many trials to accumulate.

We applied Supervised Varimax to construct task-aligned symptom composites that sharpened treatment differences. Supervised Varimax learns a small number of orthogonal symptom composites whose weights maximize between-arm separation while remaining directly interpretable as item loadings (9, 47). In STAR*D Levels 2/2A, the learned outcome indicated that hypersomnia improved more with bupropion than with venlafaxine (**Figure 1A**, right), whereas venlafaxine showed relative advantages across most other depressive symptoms (**Figure 1A**, left).

Using permutation testing that reran the full pipeline under permuted treatment assignment and controlled family-wise error rate across learned composites, we found a significant difference in the learned outcome (difference = 0.384, *p*_FWER_ = 0.007, **Figure 1C**). We summarize separation as the standardized difference in mean outcomes between arms at the final follow-up visit, similar to Cohen’s *d*; positive values indicate less improvement for bupropion. We estimated the 95% confidence intervals in **Figure 1C** by bootstrap resampling with full refitting.

We then used CO-MED as the *confirmatory* study. The prespecified confirmatory hypothesis was not that the exact composite learned in STAR*D would transport unchanged, but that the same prespecified outcome-learning procedure would recover a task-aligned endpoint capable of detecting separation between a bupropion-containing regimen and a non-bupropion strategy in an independent trial. This approach was appropriate because CO-MED differed materially from STAR*D in the treatment contrast under study: CO-MED did not compare bupropion-SR with venlafaxine-XR, but rather escitalopram plus bupropion-SR with venlafaxine-XR plus mirtazapine.

As in STAR*D, the QIDS-SR total score provided little discrimination between these combination strategies (**Figure 1E**). Because of the confirmatory hypothesis, we followed **Table 1** and learned a trial specific outcome within CO-MED using the same prespecified procedure. The learned outcome suggested that escitalopram plus bupropion was superior to venlafaxine plus mirtazapine across most symptoms, with exceptions for decreased appetite, decreased weight, and insomnia, which received relatively greater weight in favor of the mirtazapine-containing strategy (**Figure 1D**). This pattern is pharmacologically plausible given mirtazapine’s antihistaminergic and related sedative/appetite-stimulating effects (60). The learned outcome also produced clearer longitudinal separation than the total score (difference =−0.302, *p*_FWER_ =0.022, **Figure 1F**). We again obtained *p*-values by permutation testing and confidence intervals by bootstrap resampling with full refitting.

The point of this exercise is not that outcome learning retrospectively rediscovers what clinicians already know about bupropion. Rather, the underlying differential signal is real, yet broad total scores fail to detect it. By increasing sensitivity to task-relevant symptom patterns, outcome learning can recover clinically meaningful differences from individual trials. This is especially important for novel treatments, for which the clinically relevant symptom profile may be unknown and for which earlier detection of differential effects could improve trial interpretation and downstream development decisions.

## 7 Discussion

Broad symptom inventories and standardized scales remain essential for communication and cumulative science in psychiatry, but fixing a single omnibus scoring rule too early can obscure the very signals those instruments contain. When predictability or treatment responsiveness concentrates in a subset of symptoms, total scores can average away task-relevant change and produce weak prediction, null trials, and overly pessimistic interpretations. Outcome learning reframes heterogeneity from an obstacle into a design input: by prespecifying an item pool, constraints, and a machine learning algorithm, investigators can derive clinically interpretable, task-aligned targets that better match the inferential goal while preserving transparency and comparability.

These same principles also apply in confirmatory research. Once exploratory work has identified a plausible task-aligned endpoint, the key confirmatory question is not whether investigators should simply fix outcomes, but what the prespecified confirmatory hypothesis requires them to hold fixed. When the confirmatory hypothesis concerns a particular learned endpoint derived previously, that outcome should be transported and evaluated as a fixed target. When the confirmatory hypothesis instead concerns whether a prespecified outcome-learning procedure can recover a task-aligned endpoint for a given scientific question, investigators should fix the procedure and its constraints and then evaluate confirmation using relearned outcomes in the new study. With appropriate safeguards—constrained construction, end-to-end inference, multiplicity control, and external validation—outcome learning can reduce endpoint dilution without inviting *post hoc* endpoint shopping.

Finally, this agenda defines a concrete opportunity for machine learning in psychiatry. Psychiatric studies are unusually well positioned for outcome learning because commonly used questionnaires and rating scales already contain many clinically meaningful, task-relevant items. Rather than relying primarily on off-the-shelf algorithms designed for other problems, the field should continue to develop methods tailored to psychiatric measurement, with explicit constraints that preserve clinical interpretability, comparability, and external validity. The central design question is therefore not only whether to learn outcomes, but how to align the learning objective and constraints with the clinical question.

## Data Availability

Publicly available datasets were analyzed in this study. These data can be found here: https://nda.nih.gov/edit_collection.html?id=2148 (NIMH Data Archive, ID 2148); https://nda.nih.gov/edit_collection.html?id=2158 (NIMH Data Archive, ID 2158).
